# Chemical activation of Arabidopsis SnRK2.6 by pladienolide B

**DOI:** 10.1080/15592324.2021.1885165

**Published:** 2021-03-08

**Authors:** Matleena Punkkinen, Magdy M. Mahfouz, Hiroaki Fujii

**Affiliations:** a Molecular Plant Biology Unit, Department of Biochemistry, University of Turku, Turku, Finland; b Laboratory for Genome Engineering & Synthetic Biology, Division of Biological Sciences & Center for Desert Agriculture, 4700 King Abdullah University of Science and Technology, Thuwal, Saudi Arabia; c Department of Life Technologies, University of Turku, Turku,Finland

**Keywords:** Abscisic acid, chemical activator, SnRK2.6, Pladienolide B, splicing regulation, splicing inhibitors

## Abstract

Abscisic acid (ABA) is an important phytohormone mediating osmotic stress responses. SUCROSE NONFERMENTING 1 (SNF1)-RELATED PROTEIN KINASE 2.6 (SnRK2.6, also named OPEN STOMATA1 and SNF1-RELATED KINASE 2E) is central in the ABA signaling pathway; therefore, manipulating its activity may be useful to confer stress tolerance in plants. Pladienolide B (PB) is an mRNA splicing inhibitor and enhances ABA responses. Here, we analyzed the effect of PB on *Arabidopsis* SnRK2.6. PB enhanced the activity of recombinant SnRK2.6 *in vitro* through direct physical interaction as predicted by molecular docking simulations followed by mutation experiments and isothermal titration calorimetry. Structural modeling predicted probable interaction sites between PB and SnRK2.6, and experiments with mutated SnRK2.6 revealed that Leu-46 was the most essential amino acid residue for SnRK2.6 activation by PB. This study demonstrates the feasibility of SnRK2.6 chemical manipulation and paves the way for the modification of plant osmotic stress responses.

## Introduction

Osmotic stress is commonly caused by low precipitation, heat, cold, or high soil salinity and negatively affects the physiology of plants thus reducing global crop productivity. Understanding how plants regulate their responses to environmental stresses, and finding ways to modify these responses, is therefore a critical area of research to improve crop yield. If genetically modified crops continue to be rejected as human foods, one of the ways to improve plants´ responses to stress is a chemical treatment. The phytohormone abscisic acid (ABA) is central to multiple stress responses that include osmotic stress and has important roles in several developmental processes.^1–3^ ABA accumulates under osmotic stress conditions and induces stomatal closure.^4^ Therefore, treatment with ABA is considered to be a useful tool for modulating water use.^5^ However, because ABA itself induces negative effects on growth and is degraded quickly, ABA derivatives have been studied intensively.^6–8^ Alternatively, a chemical that activates specific parts of the ABA signaling could be used for improving crop stress tolerance and for studying physiological mechanisms of stress responses.

During osmotic stress responses, ABA binds to the ABA receptors PYRABACTIN RESISTANCE 1 (PYR1)/PYR1-LIKE (PYL)/REGULATORY COMPONENTS OF ABA RECEPTOR (RCAR), which interact with clade-A protein phosphatase 2 Cs (PP2Cs), resulting in the activation of the group III Sucrose Non-Fermenting1 (SNF1)-related protein kinase 2 family [SnRK2s]. ^9,10^ SnRK2s are classified into three subgroups, of which group III consists of SnRK2.2, 2.3 and 2.6, which are ABA highly activated.^11^ PYR1/PYL/RCAR-bound PP2Cs detach from kinases of the group III SnRK2s, thus allowing the kinases, which are autophosphorylated and/or phosphorylated by RAF-like Mitogen-Activated Protein Kinase Kinase Kinases (M3Ks),^12,13^ to activate and phosphorylate their downstream targets.^14^ Such targets include various transcription factors that regulate ABA-responsive gene expression.^15,16^ At the same time, group III SnRK2s also regulate stress responses in a rapid manner by phosphorylating several ion channels that are involved in stomatal opening, such as SLOW ANION CHANNEL-ASSOCIATED1 (SLAC1), POTASSIUM CHANNEL IN ARABIDOPSIS THALIANA1 (KAT1) and QUICK-ACTIVATING ANION CHANNEL1 (QUAC1).^17–20^ Besides stomatal regulation, potassium has an ameliorative effect in osmotic stress, which is crucial for maintaining water balance.^21–23^ Manipulating the activity of group III SnRK2s may therefore offer a viable option to regulate ABA signaling pathways and modulate abiotic stress responses in plants. Furthermore, since group III SnRK2s are also involved in various ABA-independent signaling pathways,^24,25^ manipulation of group III SnRK2s might also be used for the activation of these other pathways. In addition, each member of group III SnRK2s has specific roles to some extent even though they work redundantly; stomatal regulation is disrupted in the *snrk2.6* mutant while ABA responses in seed germination are attenuated in the *snrk2.2/2.3* mutant.^26–28^ Thus, activation of a specific SnRK2 could be used for selective activation of downstream signaling branches. In this study, we identify a chemical that activates SnRK2.6.

Pladienolide B (PB) was initially isolated from *Streptomyces platensis* Mer-11107 as one of seven closely-related macrolides (pladienolides A–G) and exhibited tumor-inhibiting activity as it arrests the cell cycle at the G1 and G2/M phases.^29^ PB binds to Splicing Factor 3b Subunit 1 (SF3B1) of the splicing complex and disrupts mRNA splicing, leading to the production of intron-containing mRNA precursors.^30,31^ Inhibition of splicing also occurs in plants.^32^ Notably, treating plants with PB also activates the ABA signaling pathway.^32^ Downregulation of negative regulators has been proposed as the cause of this activation: the correct splicing of *PP2C* mRNAs is disrupted in PB-treated plants, resulting in reduced activity of PP2Cs, which are negative regulators of ABA signaling whereas PB did not affect the transcript levels of other components of the ABA pathway.^32^ However, it is also possible that PB affects other components in the ABA pathway through a mechanism that does not involve splicing, since drugs often have multiple targets.^33^ This study demonstrates the effects of PB on the activity of *Arabidopsis* SnRK2.6.

## Materials and methods

### Plasmid constructs

Glutathione S-Transferase (GST)-fused SnRK2.6 was constructed by PCR with the following primers, and cloned into pGEX4T1 vector between BamHI and EcoRI sites.

2.6-F: 5´-CGGATCCATGGATCGACCAGCAGTGAGTG-3´

2.6-R: 5´-GGAATTCACATTGCGTACACAATCTC-3´

cDNAs for mutated forms of SnRK2.6 were synthesized at Eurofins Scientific SE, with nucleotide exchange listed below. The cDNAs were cloned into pGEX4T1 vector between BamHI and EcoRI sites. 61–65 NVKRE: AATGTAAAAAGGGA > GTGCGCAGAGAAGT, 44–46 NEL: AGTAATGAGCT > TCCGGAGACAA, 46 L: CTT > AAC

### Purification of recombinant proteins from E. coli

Recombinant proteins, GST-fused SnRK2.6, GST-SnRK2.2, modified GST-SnRK2.6s and GST-AREB1A, were produced in and purified from *E.coli* as described previously.^34^ For isothermal titration calorimetry, GST-fused SnRK2.6 proteins were eluted from beads with 40 mM reduced glutathione (pH 8.0) in STE solution (150 mM NaCl, 10 mM Tris-HCl pH 8.0, 1 mM EDTA). The eluted proteins were dialyzed to phosphate buffer (pH 7.4) supplemented with 150 mM NaCl at 4°C overnight.

### In vitro kinase assay

GST-fused SnRK2s were incubated with GST-fused AREB1a in 40 µl of reaction mixture (25 mM Tris pH 7.4, 12 mM MgCl_2_, 1 mM cold ATP, 5 µCi [γ-P] ATP, 1 mM DTT, 1 mM Na_2_VO_3_, and 5 mM NaF) for 30 min at 30°C in either the presence or the absence of 2 µM or indicated concentration of PB (Bioaustralis, Australia), with DMSO as control. After incubation, reactions were stopped by adding Laemmli sample buffer, and the proteins were separated on 8.5% SDS-PAGE. Radioactive bands were detected by autoradiography on the dried gel. Image Studio software (Li-Cor, USA) was used for estimating band intensities. Relative band intensities were calculated by first determining the band intensity/total protein ratios, and then normalizing these to the values of control samples. The experiments were replicated a minimum of three times.

### Modeling

The protein structure of SnRK2.6 was taken from the Protein Data Base (PDB; http://www.rcsb.org; PDB Code: 3UC4). The structure of PB was retrieved from the NCBI PubChem Database (PubChem CID: 16202130). Prediction of the protein binding sites was performed using SwissDock^35^ with the default setting.

### Isothermal titration calorimetry

Isothermal titration calorimetry was conducted with MicroCal iTC200 (Malvern, UK) according to the manufacturer’s instructions, with SnRK2.6 as the macromolecule in the cell and PB as the ligand. BSA (Sigma, USA) was used as a control macromolecule. Eluted protein samples (approximately 0.04 µg/µl) and PB (120 µM) were diluted in 4% DMSO before analysis. The following parameters were used for running the samples: Chamber volume 250 µl, number of injections 20, cell temperature 30°C, reference power 6 µCal/s, initial delay 60 s, stirring speed 1000 RPM, injection volume 0.4 µl, injection duration 0.8 s, spacing 120 s, and filter period 5 s.

## Results

### PB enhances the in vitro activity of SnRK2.6, but not SnRK2.2

**Figure 1.**
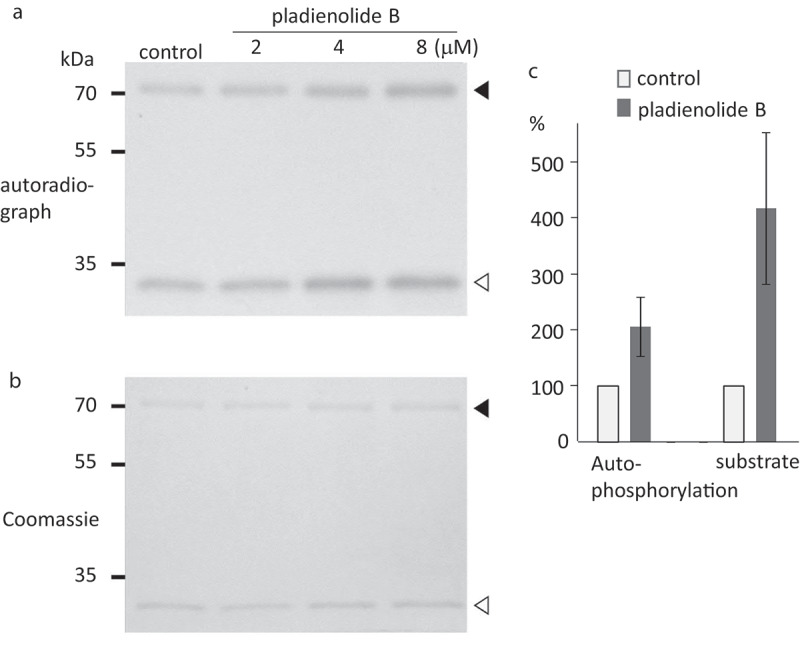
Effect of pladienolide B (PB) on the activity of recombinant SnRK2.6. Recombinant proteins were incubated with ϒ-^32^P ATP and 0, 2, 4 or 8 µM of PB. SnRK2.6 activity was estimated by exposing the gel to X-ray film and quantifying the band intensities. GST-SnRK2.6 and GST-AREB1a fragments are indicated by the black triangle and white triangle, respectively, in the autoradiograph (a) and the Coomassie-stained gel (b). (c) Relative band intensities (normalized to the level of the control condition, which was taken as 100%) were calculated for autophosphorylation (SnRK2.6) and target phosphorylation (AREB1A) after treatment with 2 µM PB (mean ± SE, *n* = 8)

To examine the effects of PB on SnRK2 family members, we first performed *in vitro* kinase assays. The activity of SnRK2.6 as determined by autophosphorylation and substrate phosphorylation was enhanced when the protein was incubated with 2 μM PB (Figure 1). In contrast, another member of group III SnRKs, SnRK2.2 showed no such response (Supp. Figure S1). The higher concentration of PB (4 and 8 μM) induced higher activity of SnRK2.6 (Figure 1(a,b)). These results indicate that PB activates SnRK2.6 *in vitro*, and that this activation cannot be generalized to other SnRK2s.

### Modeling of the interaction between PB and SnRK2.6

We observed that PB induced increased activity of SnRK2.6 in *in vitro* kinase assays, in the absence of other proteins such as PP2Cs or the splicing complex. This observation suggests that PB affects the activity of SnRK2.6 without relying on other factors, possibly by binding to the kinase directly. To examine this possibility, we performed a computational analysis of potential binding sites between SnRK2.6 and PB using SwissDock.^35^ Our analysis indicates that, the first and second highest scores for PB binding sites can be mapped to a pocket surrounded by three loops: the loop between the β2 and β3-strands (Gln-42–Leu-46), the loop between the αJ-helix and the SnRK2 box (Asn-287–Phe-295), and the loop between the β6 and β7-strands (Asp-149–Leu-156). This pocket was designated here as Site 1 (Supplemental Figure S2). The third highest-ranked location was on the opposite side of the SnRK2.6 structure from Site 1. This site, designated here as Site 2, consisted of the activation loop (Asp-160–Glu-186), the αC-helix (Ala-59–Ser-71), and the loop between the αE-helix and the β6-strand (Val-136–Lys-142; Supplemental Figure S2). These two sites were considered to be potential binding sites and were evaluated experimentally (see below).

### Mutation at the L46 residue abolishes PB-induced activation of SnRK2.6

**Figure 2.**
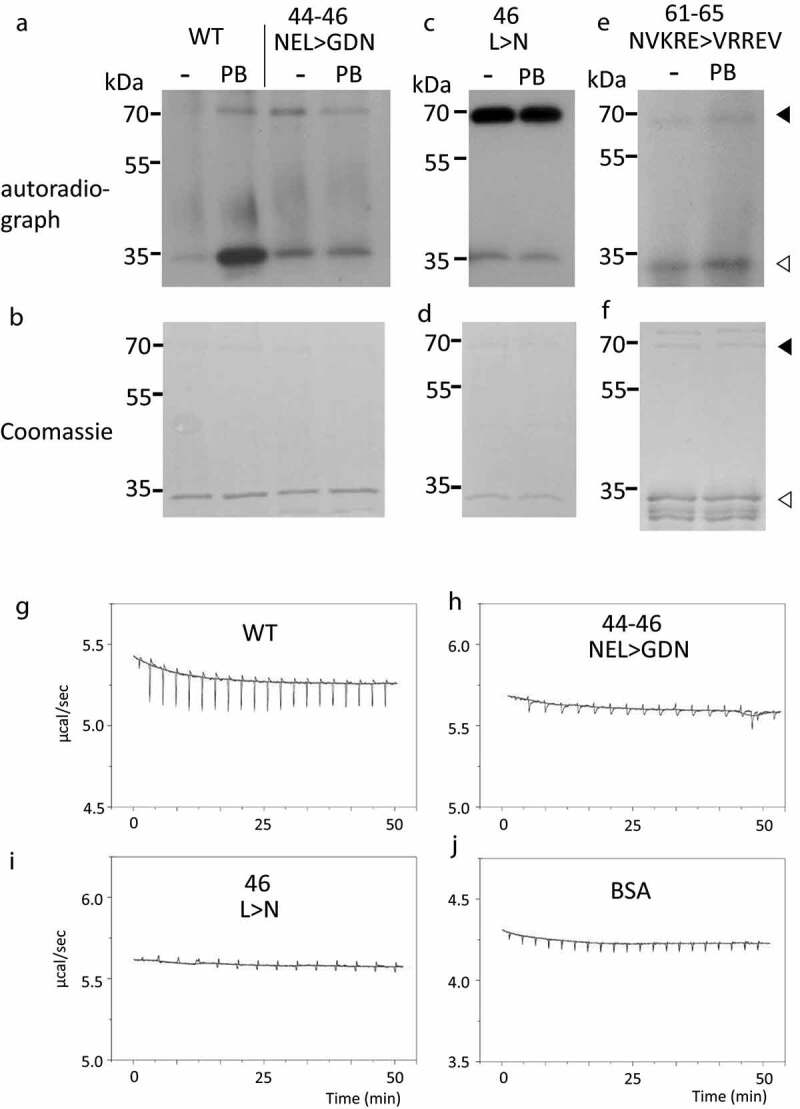
Effect of mutations on the activity of recombinant SnRK2.6. (a-f) Recombinant proteins were incubated with ϒ-^32^P ATP and 0 or 2 µM of PB. Activity of wild-type or mutated forms of SnRK2.6 were estimated by exposing the gel to X-ray film and quantifying the band intensities. GST-SnRK2.6 (wild type and mutants) and GST-AREB1a are indicated by the black triangle and white triangle, respectively, in the autoradiographs (a, c, e) and the Coomassie-stained gels (b, d, f). (g-j) Binding energy between recombinant SnRK2.6 and PB. Binding energy was measured by isothermal titration calorimetry from the interaction between PB and unmodified GST-SnRK2.6 (g), the NEL44-46GDN mutant (h), the L46N mutant (i), and BSA (j)

Next, we performed experiments with mutated forms of SnRK2.6 to identify residues that were critical for its PB-mediated activation and to evaluate the computationally calculated binding sites. GST was fused to wild-type and mutant varieties of SnRK2.6. The following mutations were tested for PB-induced activation of SnRK2.6: Asn-44–Glu-45–Leu-46 were changed to Gly-44–Asp-45–Asn46 (NEL44-46GDN), or Asn-61–Val-62–Lys-63–Arg-64–Glu65 were changed to Val-61–Arg-62–Arg-63–Glu-64–Val-65 (NVKRE61-65VRREV). We considered retaining the optimal kinase activity and the protein structure in selecting for mutated residues. PB docking to Site 1 is expected to be disrupted by the NEL44-46GDN mutations, while docking to Site 2 is predicted to be disrupted by the NVKRE61-65VRREV mutations. The NEL44-46GDN SnRK2.6 mutant was not activated by PB (Figure 2(a,b)) in our *in vitro* kinase assay. By contrast, the SnRK2.6 mutant NVKRE61-65VRREV was activated by PB (Figure 2(e,f)), indicating that Asn-61–Glu65 are not essential residues for PB-mediated activation. Site 1 was characterized further by observing the behavior of an SnRK2.6 mutant in which only Leu-46 was changed to Asn-46 (L46N) in the presence of PB. Importantly, the L46N single amino acid variant also disrupted PB-mediated activation of SnRK2.6 (Figure 2(c,d)). Taken together, these results indicate that the Leu-46 residue is critical for the activation of SnRK2.6 by PB, and suggest that Site 1 may be the binding site for PB.

### The L46N mutation disrupts the physical interaction between SnRK2.6 and PB, as detected by isothermal titration calorimetry

To further characterize the physical interaction between SnRK2.6 and PB, we performed isothermal titration calorimetry (ITC) with unmodified and mutant forms of GST-SnRK2.6. The results showed a substantial interaction between PB and wild-type GST-SnRK2.6 (Figure 2(g)). Indeed, in comparison to ITC with PB and bovine serum albumin (BSA), which is a general negative control (Figure 2(j)), we detected higher reaction energies for PB and unmodified GST-SnRK2.6. The peak heights of reaction energy decreased with repeated injections in reactions between GST-SnRK2.6 and PB, indicative of a binding reaction (Figure 2(g)), although the maximal differences between peak heights were too low for calculation of the binding constant. The small differences between peak hights are suggestive of weak interaction, as previously observed with other small molecule ligands,^36^ although low solubility of PB may affect the reaction. By contrast, the NEL44-46GDN mutant exhibited an ITC pattern that was very similar to that seen with BSA, supporting the idea that this variant does not bind to PB (Figure 2(h)). We obtained similar results with the L46N mutant (Figure 2(i)), validating this site as being responsible for binding to PB. Estimated *ΔH* were −2.84, −1.02 and −1.08 kcal/mol for the wild-type, NEL44-46GDN and L46N mutants, respectively. Taken together, these results indicate that PB can physically bind to SnRK2.6, albeit weakly, and that Leu-46 is essential for this binding.

## Discussion

The regulation of SnRK2.6 activity by protein phosphatases has been well studied; our results show that SnRK2.6 activity can also be affected chemically, opening further possibilities for controlling its function. According to structural models and experiments with mutated forms, PB may bind to SnRK2.6 via Site 1 (Figure 2 and supplemental Figure S2). The allosteric Site 1 is located on the opposite side of the reaction center, and the interaction between SnRK2.6 and bound PB may induce conformational changes that facilitate activation of the kinase. The induction of kinase activity reported here is not common to all SnRK2 members, as PB did not affect the closely related kinase SnRK2.2 (Fig. S1). From a structural point of view, the overall conformation and amino acid composition of Site 1 differs between SnRK2.2 and SnRK2.6. Specifically, SnRK2.2 and SnRK2.6 show differences in the short loop between β-strands 2 and 3, and the long “overhang” loop spanning from Ala-283–Gln-300 (in SnRK2.6). This observation further supports the hypothesis that Site 1 is the true binding site for PB on the surface of SnRK2.6.

PB may offer several advantages over plant genetic manipulation to control SnRK2.6 activity and plant responses. Although many kinases can be modified to make them constitutively active through site-directed mutagenesis to introduce phosphorylation-mimic residues in the activation loop,^37^ such mutations ruin the kinase activity of SnRK2s.^38^ By contrast, PB may allow for activation of the wild-type SnRK2.6 without having to resort to mutagenesis. Compared to genetic engineering solutions, activation by treatment with a chemical may offer a precise and conditional method to control plant responses. Furthermore, because the application of ABA has pleiotropic effects in plants, activating a single downstream kinase in the ABA signaling cascade provides a valuable avenue to dissect the underlying signaling pathway. Thus, a chemical activator is a useful tool to modulate the function(s) of SnRK2s and guide plant responses.

Despite these intriguing results, further modification of PB will be required before it can be applied to plants, since unmodified PB inhibits mRNA splicing and therefore harms plant growth.^32^ One important direction for the future development of PB as a control agent of plant responses to ABA-mediated abiotic stresses will involve the design of a PB analog that no longer interacts with the spliceosome but maintains its interaction with SnRK2.6. Since the interaction between PB and the spliceosome requires the presence of the two methyl groups in the PB side chain,^39,40^ one avenue worth investigating is whether analogs carrying modifications at these two positions will still activate SnRK2.6.

Together, these results demonstrate that PB activates the kinase SnRK2.6, allowing a deeper understanding of plant stress signaling pathways and offering new potential methods for the development of stress-tolerant crops. Screening for drugs that specifically modulate SnRKs and subsequently engineering SnRKs variants for better responses via CRISPR-directed mutagenesis may enable us to engineer crops for chemical-induced stress tolerance.^41–43^ Coupling chemical genomics and CRISPR based gene editing and directed evolution will revolutionize our ability to engineer crops of the future.^44^

## Supplementary Material

Supplemental Material
