# Supplementary material for: Chemical activation of Arabidopsis SnRK2.6 by pladienolide B
Source: Plant Signal Behav. 2021 Mar 8;16(5):1885165. doi: 10.1080/15592324.2021.1885165 (PMC8078514; doi:10.1080/15592324.2021.1885165)
Supplement: Supplemental Material [file KPSB_A_1885165_SM8880.pdf]

Supplemental Figure 1

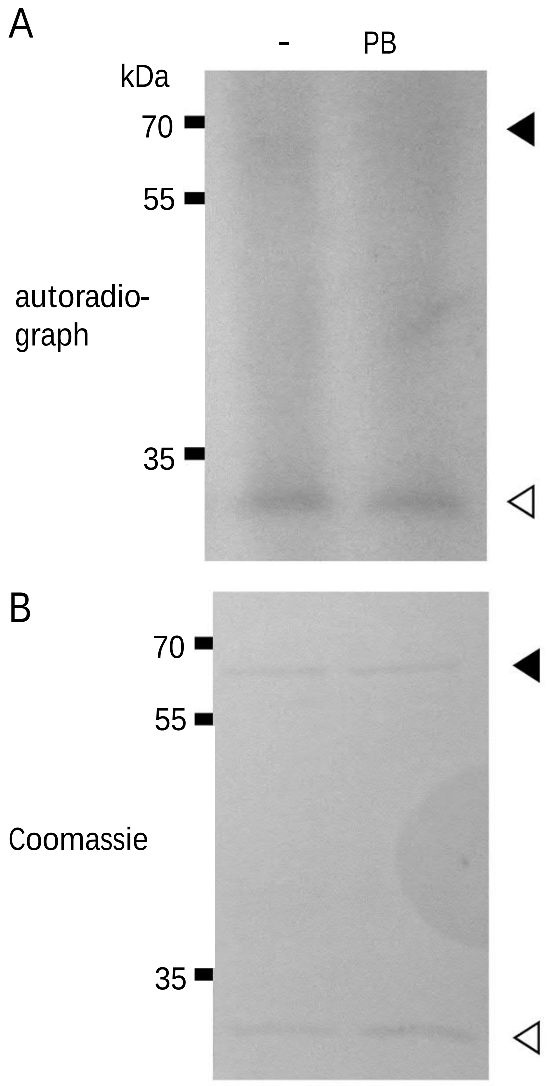

Effect of PB on the activity of recombinant SnRK2.2.
Recombinant proteins were incubated with γ-^{32}P ATP and 0 or 2 μM of PB . SnRK2.2 activity was estimated by exposing the gel to X-ray film. The positions of GST-SnRK2.2 and GST-AREB1A are indicated by the black triangle and white triangle, respectively, in the autoradiograph (A) and the Coomassie-stained gel (B).

Supplemental Figure 2

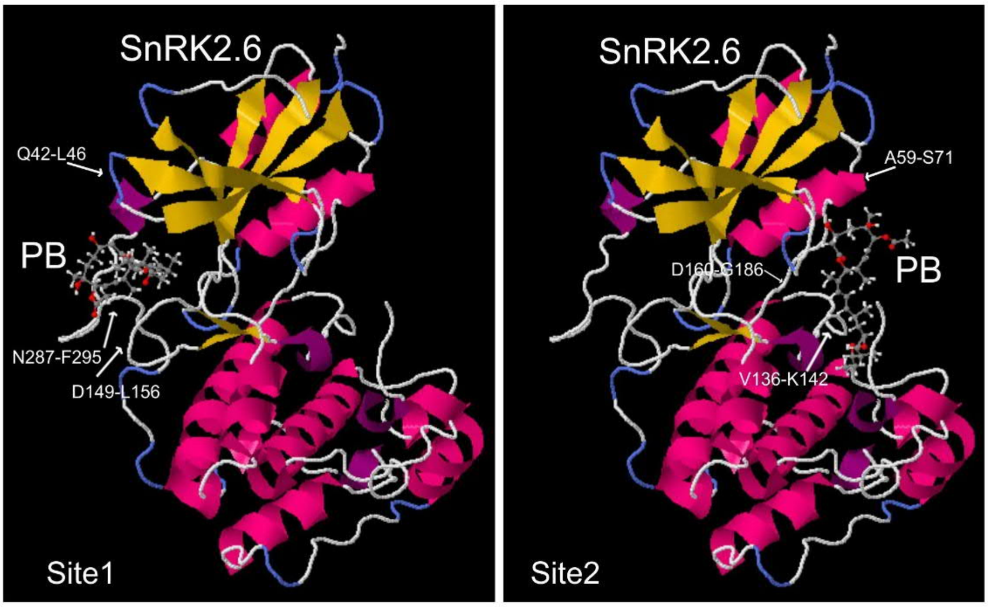

Modeling the interaction between SnRK2.6 and PB.
The highest-scoring binding model (Site 1: left) and the highest scoring binding model outside the pocket of Site 1 (Site 2: right).
